# A new partially hydrolyzed whey-based follow-on formula with age-adapted protein content supports healthy growth during the first year of life

**DOI:** 10.3389/fped.2022.937882

**Published:** 2022-09-28

**Authors:** Claude Billeaud, Latif Adamon, Hugues Piloquet, Nicholas P. Hays, Lénaïck Dupuis, Isabelle Metreau, André Léké

**Affiliations:** ^1^CIC Pédiatrique 1401 INSERM, Centre d’Investigation Clinique Bordeaux, Bordeaux, France; ^2^Department of Neonatology, Centre Hospitalier Universitaire de Caen, Caen, France; ^3^Child Chronic Disease Service, Centre Hospitalier Universitaire de Nantes, Nantes, France; ^4^Nestlé Product Technology Center – Nutrition, Vevey, Switzerland; ^5^Nestlé Clinical Research Unit, Lausanne, Switzerland; ^6^Biofortis – CIC, Saint-Herblain, France; ^7^Neonatal Medicine and Intensive Care, Centre Hospitalier Universitaire Amiens Picardie, Amiens, France

**Keywords:** infant formula, follow-on formula, protein, growth, tolerance, partially-hydrolyzed, France

## Abstract

**Background:**

Standard infant formulae often have higher protein content than breastmilk in order to compensate for potentially lower digestibility; excess protein intake may promote adverse effects later in life. A new partially hydrolyzed whey-based (pHF-W) follow-on formula (FoF) with age-adapted protein content was evaluated for growth and gastrointestinal (GI) tolerance in healthy infants.

**Methods:**

Formula-fed (FF) infants (*n* = 108) received standard pHF-W formula (1.9 g protein/100 kcal) from enrollment (age ≤ 30 days) until age 120 days followed by new pHF-W FoF (1.6 g protein/100 kcal) until 360 days. Weight gain velocity (WGV) (mean daily WG from enrollment to age 180 days) was compared to WHO growth standards and a breastfed (BF) reference group (*n* = 86) (non-inferiority margin –3 g/day). GI tolerance was assessed using a validated questionnaire (scale range 13−65).

**Results:**

WGV in FF infants (mean ± SD 24.0 ± 4.4 g/day) was non-inferior to BF (23.7 ± 3.9 g/day) and WHO standards (all *p* ≤ 0.013). Weight-for-age, length-for-age, weight-for-length, and head circumference-for-age z-scores of FF infants were not significantly different from BF at any timepoint. Symptoms of GI intolerance were low (≤23) at all timepoints and similar between groups.

**Conclusion:**

A new pHF-W FoF with age-adapted protein content fed sequentially after standard pHF-W infant formula is safe, well-tolerated, and promotes a healthy growth pattern consistent with BF infants and WHO standards during the first year of life.

**Clinical trial registration:**

[https://clinicaltrials.gov/], identifier [NCT03276663].

## Introduction

Healthy infants experience high rates of physical growth during the first 12 months of life, with average gains in weight and length of 140−200 g and 1.5−2.5 cm per week, respectively, from birth to age 6 months, which taper to 85−140 g and 1 cm per week from 6 to 12 months (1, 2). In parallel to these changes in growth velocity, the total protein content of breastmilk also gradually decreases over time, from approximately 2.0 g/100 mL immediately after birth, to 1.6 g/100 mL at 1 month, and then 1.1 g/100 mL (1.6 g/100 kcal) from about 3 months onward (3). This change in protein concentration allows breastmilk to meet the decreasing protein needs of infants during the first year of life, even when considering the increasing volume of milk intake over time (4).

Infant formulae contain more protein than breastmilk in order to provide adequate amounts of each essential amino acid required to support growth during infancy and to account for potential decreases in protein bioavailability that may occur during manufacture (5). Protein intake per kg body weight in formula-fed infants has been shown to be 55−80% greater than that of breastfed infants (6). Consequently, formula-fed infants tend to have greater weight gain in the first year of life when compared with breastfed infants (7, 8).

Rapid weight gain in infancy has been associated with childhood and adult overweight and obesity (9–12). A systematic review of 282 prospective studies evaluating risk factors for childhood overweight and obesity within the first 1,000 days of life found a consistent, positive association between accelerated infant weight gain and later childhood overweight (13). Another systematic literature review and meta-analysis of 17 studies showed significantly higher odds of overweight/obesity from childhood to adulthood among infants with a rapid weight gain (14). In line with these findings, Blake-Lamb et al. (15) identified protein-enriched formula feeding during the first 1000 days of life as one factor that can strongly increase childhood obesity risk. This was also demonstrated in a randomized trial setting, where infants fed a lower-protein infant formula and follow-on formula (1.77 and 2.2 g protein/100 kcal) in the first year of life had a lower weight at age 2 years (16) and reduced BMI at age 6 years (17) compared to infants fed higher-protein formulae (2.9 and 4.4 g protein/100 kcal).

Given this background, there have been intensive efforts to develop infant formulae with lower, more optimal, age-adapted protein concentrations to help modulate long-term obesity risk. These efforts have primarily involved formulae with intact (non-hydrolyzed) protein sources. For example, in a trial (18) of healthy infants born to mothers with a pre-pregnancy BMI > 25 kg/m^2^ and thus with increased obesity risk (19), infants were enrolled soon after birth and mothers received advice to encourage breastfeeding. Whenever mothers were unable to continue breastfeeding, an intact protein formula (1.8 g/100 kcal) was provided by the study physician/investigator. At age 3 months, predominantly formula-fed infants were randomized to receive either a lower-protein (1.65 g intact protein/100 kcal) or control formula (2.7 g protein/100 kcal) until age 12 months (18). Infants fed the lower-protein formula gained less weight between 3 and 6 months compared to infants fed the control formula, and lower body weight and decreased weight gain were observed through age 24 months (18). Another trial of infants born to obese, overweight, and normal weight mothers with a similar design reported a non-statistically significant decrease in weight gain from 3 to 6 months among infants fed a lower-protein formula (1.61 g intact protein/100 kcal) compared with those fed a control formula (2.15 g protein/100 kcal) (20).

Cow’s milk proteins in infant formulae can be partially hydrolyzed into smaller oligopeptides with reduced molecular weight, or extensively hydrolyzed into even smaller peptide fragments (21). Specific hydrolyzed whey protein formulae may reduce the risk of cow’s milk allergy (22), allergenicity (23), and atopic dermatitis (24, 25) in at-risk infants. In addition, hydrolyzed proteins may promote more favorable markers of gastrointestinal (GI) tolerance as they are more easily digested than intact proteins (26, 27). Partially hydrolyzed whey-based formulae have been recommended in some expert guidelines for non-breastfed, high-risk infants to help prevent the occurrence of atopic disease (28–31), however, results are somewhat inconsistent (32) and efficacy may depend on various clinical, economic, and cultural factors (33, 34).

Similar efforts to develop pHF-W formulae with age-adapted, lower protein concentrations have also been made, however, the existing literature is more limited, and the “lower” value that has been examined is generally higher than that evaluated for intact protein formulae. This is at least partly due to legal restrictions: the current Delegated Regulation (EU) No. 2016/127 defining the composition of infant formula and FoF requires that all protein hydrolysates used in the manufacture of these products are authorized. The authorization is based on a positive assessment by the European Food Safety Authority (EFSA) with regard to the clinically evaluated suitability of the specific protein hydrolysate (35). Currently, at the time of the preparation of this manuscript, only one protein hydrolysate has been clinically evaluated and included in the aforementioned Delegated Regulation, and the allowed protein content has been set at 1.86–2.8 g/100 kcal based on an EFSA evaluation. In this context, Ziegler et al. (36) demonstrated the suitability of this protein hydrolysate by showing that pHF-W formula with 1.9 g protein/100 kcal promoted age-appropriate infant growth in the first four months of life and was well-tolerated with incidences of flatulence, vomiting, and spitting up comparable to that of infants fed a partially hydrolyzed control formula containing 2.2 g protein/100 kcal. Non-inferior weight gain was also shown in another trial of infants fed a pHF-W (1.9 g/100 kcal) vs. extensively hydrolyzed protein formula (2.3 g/100 kcal) (37). To our knowledge, only one previous study has assessed pHF-W formula with a protein level below this range; Rigo et al. (38) showed that infants fed with pHF-W containing 1.8 g protein/100 kcal had equivalent weight gain from enrollment (≤14 days of age) to 4 months compared to infants fed pHF-W with 2.27 g protein/100 kcal.

Therefore, the aim of this study was to assess a new pHF-W follow-on formula (FoF) with a protein content (1.6 g/100 kcal) below the current EU legal minimum content, and which more closely reflects the protein content, protein quality, and amino acid profile of breastmilk from 4 to 12 months of age compared to those previously studied. It is hypothesized that this new pHF-W FoF may reduce the obesity risk associated with excess protein intake during infancy, but in a partially hydrolyzed protein format which may have other benefits/advantages. The overall objective of this trial was to assess the growth, suitability, and GI tolerance of healthy term infants fed a sequence of two pHF-W formulae with age-adapted protein concentrations (an infant formula with 1.9 g protein/100 kcal for the first 4 months of life, followed by a FoF with 1.6 g protein/100 kcal for ages 4−12 months) compared with a breastfed reference group and the World Health Organization (WHO) growth standards.

## Subjects and methods

### Study design

This prospective, open-label, interventional trial was conducted at five medical centers in France between October 2017 and June 2020. Eligible formula-fed infants received a standard pHF-W infant formula (NAN HA1^®^; 1.9 g protein/100 kcal, 67 kcal/100 mL) from enrollment (<30 days of age) through age 120 days (4 months), and a new pHF-W FoF (1.6 g protein/100 kcal, 67 kcal/100 mL) from age 120 days to 1 year of age. Infants were provided the formula orally as often as necessary as determined by the parents based on the child’s age, weight, and appetite. A group of breastfed infants served as a reference group in parallel. Breastfeeding was encouraged and lactation support *via* a lactation counselor was provided at each clinic visit for the duration of the study. From enrollment (<30 days) until age 3 months, exclusive breastfeeding was required in this reference group. From age 3 to 4 months, breastfed infants were permitted supplementation with 200 mL (1 daily bottle) of commercial formula of parent’s choice, but mothers were still encouraged by the lactation counselor to continue providing breast milk until age 6 months. Infants in both the formula-fed group and breastfed group were permitted the introduction of complementary foods beginning at 4 months of age, based on current recommendations in France (39). If breastfeeding mothers decided to stop breastfeeding despite strong encouragement and support from the lactation counselor, vouchers providing a discount on the purchase of Guigoz follow-on formula were offered in order to minimize variability in dietary intake that might have occurred if formulae were self-selected. Considering that such vouchers might influence a mother’s decision to continue breastfeeding or not, vouchers of equal value but providing a discount on the purchase of Nestlé or Guigoz infant nutrition products (excluding formula milk) were offered to mothers who did not wish to introduce formula. In addition, a standardized information sheet containing recommendations for appropriate complementary feedings was developed by the dietitian at the lead study site (CHU Bordeaux) and provided to all parents. The information sheet provided guidance for the gradual introduction of vegetables, meat/fish/eggs, fruits, starches, and fats, and included general recommendations on cooking method/texture, portion size, as well as the timing (e.g., not before the 5th month of life) and order of introduction (e.g., ensuring the infant accepts the taste of vegetables before offering fruits). Raw vegetables were not recommended to be introduced before the age of 1 year due to choking risk.

Clinic visits were conducted at enrollment and ages 1, 2, 4, 5, 6, 9, and 12 months. Phone calls were conducted at age 3, 7.5, 10.5, and 13 months. At the baseline visit, demographics, parent and household characteristics, medical history, and anthropometric measures (body weight, length, head circumference) were collected and a clinical examination was performed. At all subsequent visits, a clinical examination was conducted, and anthropometry was obtained. Lactation counseling was provided at each clinic visit for mothers of breastfed infants. Formula intake was monitored during each clinic visit and phone call. Dietary intake was assessed using a single 24-h recall conducted by a dietitian or other trained person at clinic visits at 4, 5, 6, 9 and 12 months of age as well as the 7.5- and 10.5-months phone call (results from the assessments conducted by phone are not presented due to potential data quality issues). GI tolerance was assessed using the Infant Gastrointestinal Symptom Questionnaire (IGSQ) (40) during clinic visits at 4, 5, and 6 months of age. Blood samples were collected only for formula-fed infants at 4 and 6 months of age. Parent-reported adverse events (AE) were recorded throughout the study.

This study was approved by a central research Ethics Committee (Comité de Protection des Personnes SUD-EST IV, Lyon, France) and the French national health authority (Agence Nationale de Sécurité du Medicament et des Produits du Santé). For all infants, written informed consent was obtained from the parents or legal guardian(s) (hereafter, “parents”) prior to any study-related procedures. The trial was registered on clinicaltrials.gov as NCT03276663 and performed in accordance with the International Conference on Harmonization guidelines for Good Clinical Practice and the provisions of the Declaration of Helsinki and its amendments.

### Participants

Healthy term (born between 37 and 42 weeks gestation) infants aged ≤30 days at enrollment were eligible for enrollment. Age at enrollment was originally intended to occur ≤14 days after birth, but this was later amended to ≤30 days due to challenges in enrolling exclusively formula-fed infants; many mothers had not yet decided whether to maintain breastfeeding or exclusively formula-feed within the first 2 weeks after their infant’s birth. Eligible infants weighed between 2500 and 4200 g at birth and were born to mothers with a pre-pregnancy BMI ≥ 18.5 and <28 kg/m^2^. Mothers of infants eligible for the formula-fed group had independently elected not to breastfeed before study enrollment, though infants were permitted to have received breastmilk prior to enrollment. Mothers of infants eligible for the breastfed reference group were required to intend to breastfeed through six months of age. Infants with cognitive or physical developmental disorders that would affect absorption or growth and those whose mothers had hormonal or metabolic diseases or used illicit drugs (e.g., marijuana, cocaine, amphetamines, or heroin), alcohol (>3 drinks per week), smoked >10 cigarettes per day during pregnancy, or were not expected to comply with the study protocol were excluded.

### Study formulae

The protein hydrolysate and formulae manufactured with the protein hydrolysate were manufactured under ISO 9001:2015 (quality management systems) and ISO 22000:2005 (food safety management systems). Both formulae were 100% partially hydrolyzed whey protein-based and were compliant to Annex I and II of Delegated Regulation (EU) 2016/127 (35), except for the protein amount of the FoF (1.6 g/100 kcal instead of the legal minimum amount of 1.86 g/100 kcal; see Supplementary Table 1). The protein hydrolysate used for the manufacturing of both the infant formula and FoF was identical (only the protein content differed and as a consequence the amino acid profile per 100 kcal as well) (35). The formulae were provided as powders and packaged in 800 g tins with scoops. Parents were instructed to reconstitute the formulae according to instructions on the label.

### Outcome measures

The primary outcome was weight gain velocity, measured in grams/day (g/day) from enrollment through age 180 days (6 months of age). Secondary outcomes included weight gain velocity from 4 to 6 months of age, other anthropometric measures (weight, length, head circumference, BMI, and corresponding WHO *z*-scores), biomarkers of protein status, GI tolerance, dietary intake, compliance, and incidence of allergy symptoms and allergy diagnosis. Anthropometric outcomes were measured using standard procedures. Infants were weighed without clothing or diaper on a calibrated electronic weighing scale to the nearest 1 g. Recumbent length was measured using a standardized length board to the nearest 0.1 cm. Head circumference was measured to the nearest 0.1 cm using a standard non-elastic plastic-coated measuring tape. Corresponding z-scores for each measure were calculated using the WHO Child Growth Standards (41).

Gastrointestinal tolerance was assessed using the validated IGSQ which is comprised of 13 questions covering 5 symptom domains (stooling, spitting-up/vomiting, crying, fussiness, and flatulence) (40). The questionnaire was translated into French using a standard methodology which included linguistic validation, in accordance with the Principles of Good Practice for the translation and cultural adaptation of patient-reported outcome measures (42). Each question asks about the week prior to the visit and is scored between 1 (indicating absence or minimal amount of the symptom) and 5 (indicating a high frequency, duration, or intensity of the symptom). Composite IGSQ scores can range from 13 to 65 with lower scores representing lower GI symptom burden.

Biomarkers of protein and amino acid status were measured in the formula-fed group only. Since these parameters can be interpreted in relation to standard laboratory normal ranges, we felt that it would be unnecessarily burdensome to perform blood sampling in the breastfed infants. As the infant formula has already been demonstrated to provide suitable nutrition and promote growth similar to that of breastfed infants during the first four months of age or longer (36, 43–46), protein biomarkers (blood urea nitrogen [BUN], serum albumin and pre-albumin) were collected at age 4 months just before the switch to the lower-protein FoF. Protein markers were measured again at age 6 months, corresponding to the end of the period in which the formula was still the primary source of nutrition, along with plasma amino acids (in a subset of the first 30 consented infants only). Results were compared against laboratory reference ranges. Dietary intake and feeding compliance were measured *via* feeding questionnaires at each clinic visit and phone call. Dietary intake was assessed as solid food and liquid consumption (other than study formula) over a typical day and converted into macronutrients and micronutrients using Nutrilog software (Marans, France). Compliance was determined by the reported number of days when study formula intake was interrupted. AEs (including pre-specified events of interest milk allergy, cow’s milk intolerance, lactose intolerance, eczema, atopic dermatitis, dry skin, seborrheic dermatitis, dermatitis, urticaria, and skin reaction) were recorded at each visit and categorized using Medical Dictionary for Regulatory Activities (MedDRA) version 19.

### Statistical methods

All statistical analyses were conducted using Statistical Analysis System software (SAS Institute, Cary, NC, United States) version 9.4. The sample size was calculated based on the primary outcome of weight gain velocity (g/d). The non-inferiority margin was set at –3 g/day for boys and girls against both the WHO standards and the breastfed reference group according to guidelines from the American Academy of Pediatrics (47). Sample size calculations were performed separately by sex and by comparison group (WHO standards and breastfed infants). A Bonferroni multiplicity correction was applied to account for the two comparison groups. In a previous trial of high vs. low protein content infant formula conducted in France (48), boys grew slightly less than the WHO standard (–1.33 g/day, standard deviation [SD] 4.48), while girls grew at approximately the same rate (SD 3.66). Assuming an SD of 4.48 for boys and 3.66 for girls and a dropout rate of 10%, 115 formula-fed infants (80 boys and 35 girls) and 85 breastfed infants (50 boys and 30 girls) were required to demonstrate non-inferiority at a significance level of 1.25% (i.e., adjusted *p*-value < 0.025 due to multiplicity correction and one-sided test) and a power of 80%. For secondary outcomes, *p*-values < 0.05 were considered significant.

Difference in weight gain velocity (g/day) from enrollment through age 6 months was compared with the WHO median using ANCOVA with the difference between baseline weight (weight at enrollment) and corresponding WHO standard value and study center as independent variables. The comparison with the breastfed reference group was analyzed similarly with baseline weight, feeding group, and study center as independent variables. Analyses were conducted for each sex separately and together as a supportive analysis including sex as an independent variable. Weight gain velocity was considered non-inferior for the formula-fed group if the lower bound of the one-sided confidence interval (CI) of the difference compared with either the WHO median or the breastfed reference group was above the non-inferiority margin of –3 g/day in both boys and girls. Similar analyses were performed for length and head circumference gain velocity. Differences in anthropometry measures between the formula-fed group and the WHO median were analyzed with linear mixed models (LMM) including the baseline parameter, visit, sex, the interaction between sex and visit, and study center as fixed effects and a subject-specific random effect to account for repeated measures. The difference with the breastfed group for each parameter and corresponding *z*-score was analyzed in a similar fashion. A non-inferiority margin of –0.5 with a two-sided *p*-value was also used to help interpret differences in *z*-scores (49).

Summary statistics (mean, SD) were calculated for BUN, serum albumin, pre-albumin, and the 24 collected amino acids. Values were compared to standard ranges provided by the laboratory and the number of values below the normal range was provided by sex and visit. Difference in log-transformed total IGSQ score was analyzed using LMM including feeding regimen, sex, and study center as fixed effects and a subject-specific random effect to account for repeated measures. The incidence of AEs and allergy-related AEs was compared between groups as the number of subjects with at least one event using Fisher’s exact test and odds ratios.

The intent-to-treat (ITT) population included all subjects enrolled in either the formula-fed or breastfed group. The safety (SAF) population was comprised of all subjects from the ITT population who received at least one dose of the formula or were breastfed. The full analysis (FAS) dataset included all subjects from the SAF population with data from at least one post-enrollment visit. The per-protocol (PP) population included all subjects from the FAS population after excluding those with major protocol deviations believed to impact the primary analysis, such as interruption of study formula ≥5 consecutive days before age 4 months or ≥7 consecutive days from age 4−6 months, consumption of ≥4 teaspoons of complementary foods before age 4 months, or the 4-month clinic visit occurring outside of the visit window (113–127 days). The primary and secondary analyses were conducted on the PP and FAS populations and the incidence of AEs was conducted in the SAF population.

## Results

All screened infants were able to be enrolled in the trial and comprised the ITT population (*n* = 194, comprised of 72 formula-fed boys, 36 formula-fed girls, 51 breastfed boys, and 35 breastfed girls). During study conduct, average weight gain velocity data were monitored to assess agreement with sample size assumptions. It was observed that boys on average were growing with approximately the same velocity as the WHO standard and girls were growing a bit faster, with SDs in both groups similar to those anticipated, suggesting that the original sample size was likely overestimated. Therefore, it was decided in June 2019 to stop enrollment.

**FIGURE 1:**
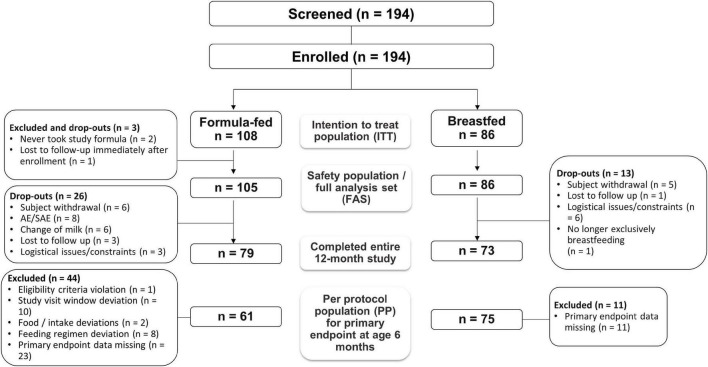
Flow of study participants.

The flow of subjects through the trial is shown in Figure 1. Two formula-fed infants who never received the study formula and one formula-fed infant lost to follow-up the day of enrollment were excluded leaving 191 in the SAF and FAS populations. Thirty-nine subjects (26 formula-fed infants; 13 breastfed infants) dropped out of the study, primarily without an explanation (6 formula-fed; 5 breastfed) or due to an AE (*n* = 8) or change of milk (*n* = 6) among formula-fed infants. One breastfed infant dropped out after premature discontinuation of exclusive breastfeeding. Overall, 152 infants (79 formula-fed; 73 breastfed) completed the study; after excluding infants with major protocol deviations expected to impact weight gain, 136 infants (61 formula-fed; 75 breastfed) comprised the PP population.

Infants’ weight and length at birth were similar between the formula-fed and breastfed groups (Table 1). Two-thirds of formula-fed infants and 60% of breastfed infants were male (this imbalance between sexes was by design, given that the study was powered for each sex separately). At enrollment, anthropometric measures were comparable between groups. Parents of infants in the breastfed group generally had higher levels of education than those of the formula-fed group. Further, both fathers and mothers of infants in the formula-fed group had higher rates of smoking than breastfed infants’ parents.

**TABLE 1:** Characteristics of study infants, by feeding group (ITT population).

	Formula-fed infants (*n* = 108)	Breastfed infants (*n* = 86)
Sex, *n* (%) male	72 (66.7%)	51 (59.3%)
**Anthropometric measures at birth**		
Weight (kg)		
Female	3.21 ± 0.34	3.32 ± 0.42
Male	3.41 ± 0.40	3.47 ± 0.33
Total	3.34 ± 0.39	3.41 ± 0.37
Length (cm)		
Female	48.86 ± 1.60	48.97 ± 1.60
Male	49.89 ± 2.23	50.34 ± 1.83
Total	49.55 ± 2.10	49.78 ± 1.86
**Anthropometric measures at enrollment**		
Weight (kg)		
Female	3.11 ± 0.34	3.17 ± 0.40
Male	3.40 ± 0.44	3.33 ± 0.32
Total	3.30 ± 0.43	3.26 ± 0.36
Length (cm)		
Female	49.09 ± 1.62	49.43 ± 1.40
Male	50.48 ± 2.39	50.43 ± 1.78
Total	50.02 ± 2.25	50.02 ± 1.70
Head circumference (cm)		
Female	34.37 ± 0.88	34.25 ± 1.01
Male	35.06 ± 1.39	35.20 ± 1.05
Total	34.83 ± 1.28	34.81 ± 1.13
**Number of days of breastfeeding before enrollment**	3 ± 2.7	4 ± 3.1
**Father’s age at enrollment (y)**	32 ± 5.9	34 ± 5.1
**Mother’s age at enrollment (y)**	30 ± 4.8	32 ± 4.7
**Father’s highest level of education, *n* (%)**		
Middle school	8 (7.4%)	0 (0%)
High school/professional school	45 (41.7%)	25 (29.1%)
Bachelor’s degree	27 (25%)	20 (23.3%)
Master’s degree	13 (12%)	23 (26.7%)
Doctorate	5 (4.6%)	13 (15.1%)
Other	6 (5.6%)	4 (4.7%)
Missing	4 (3.7%)	1 (1.2%)
**Mother’s highest level of education, *n* (%)**		
Middle school	7 (6.5%)	2 (2.3%)
High school/professional school	45 (41.7%)	16 (18.6%)
Bachelor’s degree	35 (32.4%)	25 (29.1%)
Master’s degree	8 (7.4%)	23 (26.7%)
Doctorate	7 (6.5%)	15 (17.4%)
Other	6 (5.6%)	5 (5.8%)
**Fathers who smoked at enrollment, *n* (%)**	51 (47.2%)	29 (33.7%)
**Mother’s smoking history**		
Never smoker, *n* (%)	54 (50%)	57 (66.3%)
Smoked during pregnancy, *n* (%)	24 (22.2%)	6 (7%)
Number of cigarettes smoked during pregnancy	4 ± 2.3	6 ± 2.8
Smoked post-pregnancy, *n* (%)	18 (16.7%)	5 (5.8%)
Number of cigarettes smoked post-pregnancy	3 ± 2.2	4 ± 1.8

Values are mean ± standard deviation unless otherwise noted.

### Growth

From enrollment to 6 months of age (180 days), infants in the formula-fed group had similar weight gain velocity compared to infants in the breastfed group for both girls (23.1 ± 4.5 vs. 22.1 ± 3.7 g/day) and boys (24.6 ± 4.4 vs. 24.8 ± 3.8 g/day) in the PP population (Table 2). The adjusted mean difference between groups was 0.23 g/day (one-sided 98.75% CI: −2.18) for girls and 0.62 g/day (−1.64) for boys. Similar results were observed in the FAS population (Table 2). As the lower boundaries of the confidence intervals for both boys and girls were higher than the non-inferiority margin of –3 g/day, the adjusted weight gain velocity of formula-fed infants demonstrated non-inferiority to that of breastfed infants. When compared to the WHO median values, the adjusted difference in weight gain velocity in formula-fed infants was 1.99 g/day (–0.26) for girls and 0.47 (–2.32) for boys, which also demonstrated non-inferior growth between formula-fed infants and the WHO median. Based on results from a prior French infant formula trial (48), the sample size calculation accounted for an anticipated lower growth rate in boys vs. girls compared to the WHO median (–1.33 vs. –0.06 g/day, respectively); however, in the current trial, both boys and girls grew at more comparable rates to the WHO median between enrollment and age 6 months (+0.02 and +0.55 g/day, respectively).

**TABLE 2:** Weight gain velocity (g/d) from 0 to 6 months (i.e., enrollment to 180 days).

Analysis set	Group	Sex	Mean ± SD weight gain velocity (g/d)	Estimated* treatment effects for weight gain velocity			
				Comparison	Estimated difference (g/d)	One-sided 98.75% CI	Adjusted *P*-value (non-inferiority)
PP (*n* = 136)	Formula-fed	Female	23.08 ± 4.45	Formula-fed vs. WHO median	1.99	(−0.26)	<0.001
		Male	24.64 ± 4.38	Formula-fed vs. WHO median	0.47	(−2.32)	0.013
	Breastfed	Female	22.14 ± 3.66	Formula-fed vs. breastfed	0.23	(−2.18)	0.006
		Male	24.79 ± 3.81	Formula-fed vs. breastfed	0.62	(−1.64)	<0.001
FAS (*n* = 157)	Formula-fed	Female	23.11 ± 4.25	Formula-fed vs. WHO median	2.06	(−0.01)	<0.001
		Male	25.16 ± 4.19	Formula-fed vs. WHO median	0.55	(−1.24)	<0.001
	Breastfed	Female	22.14 ± 3.66	Formula-fed vs. breastfed	0.33	(−1.94)	0.003
		Male	24.79 ± 3.81	Formula-fed vs. breastfed	0.68	(−1.23)	<0.001

*Adjusted for baseline weight and study center; PP, per-protocol; FAS, full analysis set.

**FIGURE 2:**
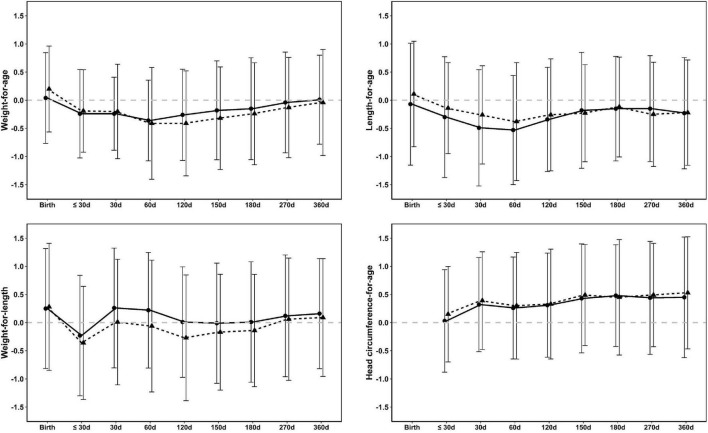
Mean ± SD weight-for-age, weight-for-length, length-for-age, and head circumference-for age *z*-scores from birth to 12 months (360 days) in formula-fed (circles, solid line) and breastfed infants (triangles, dashed line) in the FAS population. The dashed gray line represents the WHO median (36).

The growth rate in formula-fed infants between age 4 and 6 months, the period in which the lower-protein FoF was used and intake from complementary foods was still minimal, was demonstrated to be non-inferior to the breastfed group (adjusted mean difference –0.47 g/day, 95% CI: –2.16;1.23) and significantly above the WHO median (1.58 g/day, 95% CI: 0.07; 3.09). No significant differences were observed between formula-fed and breastfed infants with regard to weight gain (g/d), length gain (cm/w), or head circumference gain (cm/w) assessed over the entire study interval (baseline to age 1 year) (Table 3). *Z*-scores for weight-for-age (WAZ), length-for-age (LAZ), weight-for-length (WLZ), and head circumference-for-age (HCAZ) at each visit during the study period tracked closely with the WHO reference for both groups (Figure 2) and were similar between formula-fed and breastfed infants. WAZ scores were not significantly different than the WHO median at any time other than 2 months (difference with WHO: –0.26; 95% CI: –0.48; –0.04). LAZ, WLZ, and BMI-for-age *z*-scores were only significantly different than the WHO median at one timepoint each. HCAZ scores of formula-fed infants were significantly higher than the WHO median at 1 month and between 4 and 12 months, but differences between formula-fed and breastfed infants were not significant at any time point.

**TABLE 3:** Mean ± SD growth velocity from enrollment to study end (i.e., 12 months of age) by feeding group and sex (FAS population).

	Formula-fed infants			Breastfed infants			*P*-value*
	Males (*n* = 52)	Females (*n* = 27)	Total (*n* = 79)	Males (*n* = 42)	Females (*n* = 31)	Total (*n* = 73)	
Weight gain (g/d)	17.51 ± 2.35	16.59 ± 2.41	17.20 ± 2.40	17.58 ± 2.69	16.13 ± 2.65	16.97 ± 2.75	0.589
Length gain (cm/week)	0.48 ± 0.05	0.48 ± 0.04	0.48 ± 0.04	0.48 ± 0.04	0.47 ± 0.04	0.47 ± 0.04	0.246
Head circumference gain (cm/week)	0.22 ± 0.03	0.22 ± 0.02	0.22 ± 0.03	0.23 ± 0.03	0.22 ± 0.02	0.23 ± 0.03	0.711

*p-value for difference between feeding groups, adjusted for baseline growth parameter, sex, and study center.

### Gastrointestinal tolerance

**FIGURE 3:**
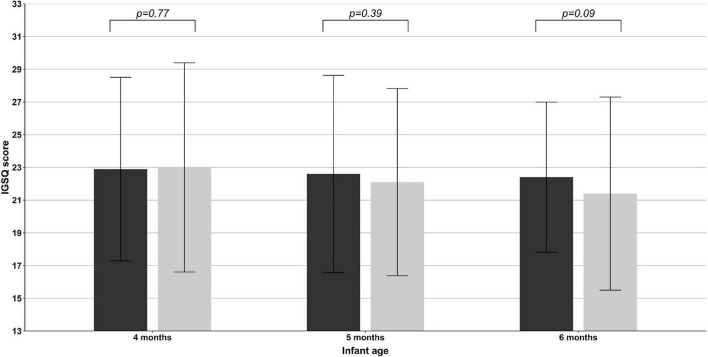
Mean ± SD Infant Gastrointestinal Symptom Questionnaire (IGSQ) scores at infant age 4, 5, and 6 months in formula-fed (black bars) and breastfed infants (gray bars) in the FAS population.

Infant Gastrointestinal Symptom Questionnaire scores ranged from 13 to 45 across all infants, with lower scores indicating less GI symptom burden. Adjusted mean scores decreased from age 4−6 months in both formula-fed infants (21.3, 21.0, and 20.8 at 4, 5, and 6 months, respectively) and breastfed infants (21.1, 20.3, and 19.6). No significant difference between groups was observed for IGSQ scores at any visit (Figure 3), indicating similar GI tolerance between infants fed the new FoF and breastfed infants. Notably, adjusted mean IGSQ scores were all below 23, which is the threshold indicative of GI discomfort (40).

### Biomarkers of protein status and amino acids

Protein biomarker levels for the formula-fed infants are shown in Supplementary Table 2. While the proportion of infants with lower-than-normal BUN levels increased from age 4 to 6 months, the mean values were similar across visits (4 months: 2.69 ± 0.59 mmol/L; 6 months: 2.40 ± 0.79 mmol/L). Concentrations of all essential amino acids were at or above the normal lower limit at 6 months of age apart from valine (2 values [7%]).

### Dietary intake from complementary foods and liquids

Total energy intake (kcal/day) from weaning foods and liquids (i.e., not including breastmilk or study formula) was similar between breastfed and formula-fed infants (Supplementary Table 3). The amount of protein consumed per day from complementary foods increased throughout the study period from about 2 g/day at 5 months to 20 g/day at 12 months. The proportion of total energy intake comprised by protein increased for both groups from about 9% at 5 months to 14% at 12 months. Fruit puree was the most commonly consumed complementary food from 4 to 6 months of age and 6−12 months of age, representing 18% and 16%, respectively, of the complementary foods usually consumed over 24 h. The next most commonly consumed foods from 4 to 6 months were vegetable puree (12% of foods consumed), cooked vegetables (12%), cereals (11%), and milk dessert (7%); and from 6 to 12 months, infant cooked dish (e.g., vegetable dish with starch and meat, fish, or cream; 11%), milk dessert (10%), cereals (8%), and cooked vegetables (7%).

### Feeding patterns

The amount of formula consumed by infants increased over the study period (1 month: 111 ± 19 mL/feeding occasion [mean ± SD]; 2 months: 136 ± 24 mL; 4 months: 174 ± 33 mL; 6 months: 179 ± 35 mL; 12 months: 218 ± 44 mL). For breastfed infants, the average intake of expressed milk consumed per feeding occasion increased (1 month: mean 83 ± 40 mL; 2 months: 99 ± 40 mL; 4 months: 150 ± 44 mL; 6 months: 155 ± 35 mL; 12 months: 169 ± 67 mL), though the number of breastfed infants who received expressed milk decreased from 15 to 5 over the study period. The average minutes of breastfeeding per feeding occasion were generally stable during the first 4 months (1 month: mean 21 ± 10 min; 2 months: 18 ± 9 min; 3 months: 20 ± 10 min; 4 months: 16 ± 7 min). Thirty-one infants in the formula-fed group definitively stopped formula-feeding over the study period; 19 of these had a major protocol deviation associated with feeding compliance. The majority (77%) of mothers in the breastfed group were still breastfeeding as of the 6-month clinic visit, and there were no reports of unauthorized concomitant diets that were considered major protocol deviations, indicating good compliance with the study protocol in the breastfed group. Approximately 72% of formula-fed infants and 35% of breastfed infants took a commercial formula other than the study formula at least once during the trial.

### Safety

Overall, the proportion of subjects with at least one AE was 92.4% (*n* = 97) in the formula-fed group and 77.9% (*n* = 67) in the breastfed group over the full study period (Table 4). From 0 to 4 months (i.e., when the study infant formula was fed), the proportion of subjects with at least one AE was much lower in both groups (formula-fed: 9.5% [*n* = 10] and breastfed: 11.6% [*n* = 10]). Among formula-fed subjects who experienced an AE, 35.2% of the events were considered probably or definitely related to the study product, 3.8% were severe, and 7.6% resulted in study discontinuation. Events considered related to study product were regurgitation, constipation, lactose intolerance, and gastrointestinal disorder. Fifteen events (14.3%) in formula-fed infants and eleven events (12.8%) in breastfed infants were serious AEs, including fever, weight loss, bronchiolitis, gastroenteritis, and injury; all occurred between 4 and 12 months. Allergy-related AEs occurred in 16.2% of formula-fed infants and 18.6% of breastfed infants. From 0 to 4 months, AEs were relatively infrequent, and no differences were observed between the feeding groups for AEs by system organ class (SOC) (Table 5). From 4 to 12 months (i.e., the period when the study FoF was fed), the most frequent AEs by SOC were infections and infestations (occurring in *n* = 70 [66.7%] formula-fed infants and *n* = 56 [65.1%] breastfed infants, namely bronchiolitis, gastroenteritis, nasopharyngitis, and rhinitis) and GI disorders (occurring in *n* = 65 [61.9%] of formula-fed infants and *n* = 37 [43.0%] of breastfed infants) (Table 5). No significant difference in the incidence of AEs was observed for any SOC except for congenital, familial, and genetic disorders (*p* = 0.025) and GI disorders (*p* = 0.021), both between 4 and 12 months.

**TABLE 4:** Adverse events (AE) by feeding group and time period (SAF population).

	Formula-fed (*n* = 105)			Breastfed (*n* = 86)		
	0 to ≤4 months	>4 to 12 months	0 to 12 months	0 to ≤4 months	>4 to 12 months	0 to 12 months
Subjects with at least one AE	10 (9.5%)	97 (92.4%)	97 (92.4%)	10 (11.6%)	67 (77.9%)	67 (77.9%)
**Seriousness**						
No	10 (9.5%)	96 (91.4%)	97 (92.4%)	10 (11.6%)	67 (77.9%)	67 (77.9%)
Yes	0 (0%)	15 (14.3%)	15 (14.3%)	0 (0%)	11 (12.8%)	11 (12.8%)
**Relationship to product**						
Unrelated	9 (8.6%)	80 (76.2%)	80 (76.2%)	10 (11.6%)	67 (77.9%)	67 (77.9%)
Unlikely	1 (1.0%)	13 (12.4%)	13 (12.4%)	0 (0%)	1 (1.2%)	1 (1.2%)
Probable	0 (0%)	33 (31.4%)	33 (31.4%)	0 (0%)	2 (2.3%)	2 (2.3%)
Related	0 (0%)	4 (3.8%)	4 (3.8%)	0 (0%)	0 (0%)	0 (0%)
Severity						
Mild	9 (8.6%)	84 (80.0%)	84 (80.0%)	10 (11.6%)	62 (72.1%)	63 (73.3%)
Moderate	1 (1.0%)	46 (43.8%)	46 (43.8%)	1 (1.2%)	25 (29.1%)	25 (29.1%)
Severe	–	4 (3.8%)	4 (3.8%)	–	3 (3.5%)	3 (3.5%)
**Caused study discontinuation**						
No	10 (9.5%)	94 (89.5%)	94 (89.5%)	10 (11.6%)	67 (77.9%)	67 (77.9%)
Yes	0 (0%)	8 (7.6%)	8 (7.6%)	0 (0%)	0 (0%)	0 (0%)
Subjects with at least one allergy-related AE*	0 (0%)	17 (16.2%)	17 (16.2%)	2 (2.3%)	14 (16.3%)	16 (18.6%)

*Including milk allergy, cow’s milk intolerance, lactose intolerance, eczema, atopic dermatitis, dry skin, seborrheic dermatitis, dermatitis, urticaria, and skin reaction. Percentages are computed with respect to the number of subjects per feeding group in the Safety Analysis (SAF) population.

**TABLE 5:** Frequency of adverse events by system organ class and preferred term, by feeding group and time period.

	0 to ≤4 months					>4 to 12 months				
	Formula-fed		Breastfed			Formula-fed		Breastfed		
				
	*n* (%) with at least one event (*n* = 105)	*n* (%) events (*n* = 16)	*n* (%) with at least one event (*n* = 86)	*n* (%) events (*n* = 20)	*P-value for difference in* n *with at least one event*	*n* (%) with at least one event (*n* = 105)	*n* (%) events (*n* = 463)	*n* (%) with at least one event (*n* = 86)	*n* (%) events (*n* = 346)	*P-value for difference in* n *with at least one event*
Congenital, familial, and genetic disorders	0 (0%)	0 (0%)	0 (0%)	0 (0%)		10 (9.5%)	10 (2.2%)	1 (1.2%)	1 (0.3%)	*0.025*
Plagiocephaly	0 (0%)	0 (0%)	0 (0%)	0 (0%)		7 (6.7%)	7 (1.5%)	1 (1.2%)	1 (0.3%)	
Ear and labyrinth disorders	0 (0%)	0 (0%)	0 (0%)	0 (0%)		1 (1.0%)	1 (0.2%)	0 (0%)	0 (0%)	>*0.99*
Eye disorders	3 (2.9%)	3 (18.8%)	3 (3.5%)	3 (15.0%)	> *0.99*	18 (17.1%)	25 (5.4%)	11 (12.8%)	15 (4.3%)	*0.545*
Conjunctivitis	3 (2.9%)	3 (18.8%)	1 (1.2%)	1 (5.0%)		17 (16.2%)	23 (5.0%)	8 (9.3%)	12 (3.5%)	
Gastrointestinal disorders	5 (4.8%)	9 (56.3%)	7 (8.1%)	7 (35.0%)	*0.376*	65 (61.9%)	114 (24.6%)	37 (43.0%)	60 (17.3%)	*0.021*
Abdominal pain	1 (1.0%)	1 (6.3%)	2 (2.3%)	2 (10.0%)		20 (19.0%)	21 (4.5%)	12 (14.0%)	12 (3.5%)	
Constipation	0 (0%)	0 (0%)	3 (3.5%)	3 (15.0%)		18 (17.1%)	19 (4.1%)	7 (8.1%)	8 (2.3%)	
Diarrhea	1 (1.0%)	1 (6.3%)	0 (0%)	0 (0%)		15 (14.3%)	19 (4.1%)	8 (9.3%)	8 (2.3%)	
Regurgitation	0 (0%)	0 (0%)	0 (0%)	0 (0%)		13 (12.4%)	14 (3.0%)	4 (4.7%)	4 (1.2%)	
Teething	3 (2.9%)	4 (25.0%)	1 (1.2%)	1 (5.0%)		11 (10.5%)	15 (3.2%)	12 (14.0%)	15 (4.3%)	
General disorders and administration site conditions	0 (0%)	0 (0%)	0 (0%)	0 (0%)		14 (13.3%)	19 (4.1%)	11 (12.8%)	18 (5.2%)	>*0.99*
Pyrexia	0 (0%)	0 (0%)	0 (0%)	0 (0%)		12 (11.4%)	15 (3.2%)	9 (10.5%)	11 (3.2%)	
Immune system disorders	0 (0%)	0 (0%)	0 (0%)	0 (0%)		2 (1.9%)	2 (0.4%)	1 (1.2%)	1 (0.3%)	>*0.99*
Milk allergy	0 (0%)	0 (0%)	0 (0%)	0 (0%)		2 (1.9%)	2 (0.4%)	1 (1.2%)	1 (0.3%)	
Infections and infestations	3 (2.9%)	3 (18.8%)	4 (4.7%)	7 (35.0%)	*0.702*	70 (66.7%)	224 (48.4%)	56 (65.1%)	194 (56.1%)	>*0.99*
Bronchiolitis	0 (0%)	0 (0%)	0 (0%)	0 (0%)		22 (21.0%)	26 (5.6%)	20 (23.3%)	32 (9.2%)	
Bronchitis	0 (0%)	0 (0%)	0 (0%)	0 (0%)		12 (11.4%)	14 (3.0%)	5 (5.8%)	10 (2.9%)	
Ear infection	0 (0%)	0 (0%)	0 (0%)	0 (0%)		15 (14.3%)	22 (4.8%)	19 (22.1%)	32 (9.2%)	
Gastroenteritis	1 (1.0%)	1 (6.3%)	0 (0%)	0 (0%)		24 (22.9%)	29 (6.3%)	16 (18.6%)	19 (5.5%)	
Nasopharyngitis	1 (1.0%)	1 (6.3%)	1 (1.2%)	1 (5.0%)		27 (25.7%)	44 (9.5%)	18 (20.9%)	24 (6.9%)	
Rhinitis	1 (1.0%)	1 (6.3%)	1 (1.2%)	1 (5.0%)		25 (23.8%)	41 (8.9%)	16 (18.6%)	34 (9.8%)	
Varicella	0 (0%)	0 (0%)	0 (0%)	0 (0%)		10 (9.5%)	10 (2.2%)	9 (10.5%)	9 (2.6%)	
Injury, poisoning and procedural complications	0 (0%)	0 (0%)	0 (0%)	0 (0%)		5 (4.8%)	6 (1.3%)	4 (4.7%)	4 (1.2%)	>*0.99*
Investigations	0 (0%)	0 (0%)	0 (0%)	0 (0%)		3 (2.9%)	3 (0.6%)	0 (0%)	0 (0%)	*0.256*
Metabolism and nutrition disorders	0 (0%)	0 (0%)	0 (0%)	0 (0%)		2 (1.9%)	5 (1.1%)	2 (2.3%)	2 (0.6%)	>*0.99*
Cow’s milk intolerance	0 (0%)	0 (0%)	0 (0%)	0 (0%)		0 (0%)	0 (0%)	1 (1.2%)	1 (0.3%)	
Lactose intolerance	0 (0%)	0 (0%)	0 (0%)	0 (0%)		1 (1.0%)	1 (0.2%)	0 (0%)	0 (0%)	
Musculoskeletal and connective tissue disorders	0 (0%)	0 (0%)	0 (0%)	0 (0%)		0 (0%)	0 (0%)	1 (1.2%)	1 (0.3%)	*0.443*
Neoplasms benign, malignant, and unspecified	0 (0%)	0 (0%)	0 (0%)	0 (0%)		2 (1.9%)	2 (0.4%)	1 (1.2%)	1 (0.3%)	>*0.99*
Hemangioma	0 (0%)	0 (0%)	0 (0%)	0 (0%)		2 (1.9%)	2 (0.4%)	1 (1.2%)	1 (0.3%)	
Nervous system disorders	0 (0%)	0 (0%)	0 (0%)	0 (0%)		0 (0%)	0 (0%)	1 (1.2%)	2 (0.6%)	*0.443*
Psychiatric disorders	0 (0%)	0 (0%)	0 (0%)	0 (0%)		0 (0%)	0 (0%)	1 (1.2%)	1 (0.3%)	*0.443*
Respiratory, thoracic, and mediastinal disorders	0 (0%)	0 (0%)	0 (0%)	0 (0%)		12 (11.4%)	17 (3.7%)	12 (14.0%)	17 (4.9%)	*0.662*
Skin and subcutaneous tissue disorders	0 (0%)	0 (0%)	3 (3.5%)	3 (15.0%)	*0.085*	32 (30.5%)	35 (7.6%)	22 (25.6%)	27 (7.8%)	*0.629*
Dermatitis	0 (0%)	0 (0%)	0 (0%)	0 (0%)		1 (1.0%)	1 (0.2%)	1 (1.2%)	1 (0.3%)	
Dermatitis atopic	0 (0%)	0 (0%)	0 (0%)	0 (0%)		1 (1.0%)	1 (0.2%)	3 (3.5%)	3 (0.9%)	
Eczema	0 (0%)	0 (0%)	2 (2.3%)	2 (10.0%)		10 (9.5%)	10 (2.2%)	6 (7.0%)	6 (1.7%)	
Seborrheic dermatitis	0 (0%)	0 (0%)	0 (0%)	0 (0%)		1 (1.0%)	1 (0.2%)	1 (1.2%)	1 (0.3%)	
Surgical and medical procedures	0 (0%)	0 (0%)	0 (0%)	0 (0%)		0 (0%)	0 (0%)	2 (2.3%)	2 (0.6%)	*0.195*
Vascular disorders	1 (1.0%)	1 (6.3%)	0 (0%)	0 (0%)	>*0.99*	0 (0%)	0 (0%)	0 (0%)	0 (0%)	

## Discussion

The current trial demonstrated that pHF-W FoF with age-adapted protein concentration, fed sequentially after pHF-W infant formula, is well-tolerated and supports age-appropriate growth in healthy term infants compared with both WHO growth standards and breastfed infants. Weight gain velocity in the first six months of life was non-inferior to that of breastfed infants and the WHO standards. In addition, weight gain velocity during the 4−6 months study interval (when the lower-protein pHF-W FoF was fed and intake from complementary foods was minimal) was non-inferior to the breastfed group and above the WHO median. WAZ, LAZ, and WLZ of formula-fed infants tracked closely with those of breastfed infants and the WHO median over the entire study duration. Specifically, the model-based mean differences for these *z*-scores were small (–0.15 to 0.19 vs. WHO and –0.02 to 0.24 vs. breastfed) and the upper and lower bounds of the 95% CI for each model-based mean difference were all within ± 0.05 SD at 12 months, indicating a healthy growth pattern (49). HCAZ scores were significantly higher in formula-fed infants vs. WHO at all timepoints (model-based mean differences ranged from 0.34 to 0.51) but were not significantly different from breastfed infants. Interestingly, a systematic review of studies reporting child growth data from 55 different countries or ethnic groups showed that head circumference values varied more widely than weight or height, and in many groups, head circumference means were consistently 0.5–1.0 SD above the WHO median (50). Values in French infants at age 2 years were reported to be > 0.5 SD above the WHO median (50), which is consistent with the findings in our study. Finally, infant GI tolerance scores were low and similar between groups between 4 and 6 months, indicating a low GI symptom burden among infants fed the new pHF-W FoF. The incidence of serious or severe AEs was low in both groups, and no increased risk of allergy-related AEs was seen in formula-fed infants as compared to breastfed infants.

The results of this study for infant growth are comparable with previous studies of reduced protein infant formulae (using either hydrolyzed or intact protein sources). A trial comparing a lower-protein partially hydrolyzed infant formula (1.9 g/100 kcal) with a higher-protein extensively hydrolyzed control formula (2.3 g/100 kcal) reported non-inferior weight gain between formula groups in the first four months of life along with *z*-scores between the 15th and 85th percentiles, indicating age-appropriate growth (37). An evaluation of an experimentally modified intact whey FoF with a protein content of 1.61 g/100 kcal compared with an unmodified intact control FoF with a protein content of 2.15 g/100 kcal from 3 to 12 months of life demonstrated normal growth in both groups with a slower weight gain in the experimental group than the control (20). Normal infant growth was further demonstrated in a study comparing a reduced partially hydrolyzed protein infant formula (1.90 g/100 kcal) with a higher-protein formula (2.39 g/100 kcal) in the first four months of life (43). These studies, together with the current trial, demonstrate that infant formulae with age-adapted protein concentrations can support adequate age-appropriate growth in an infant’s first year.

The association between higher protein content and increased weight gain was shown in the European Childhood Obesity Trial, where infants fed lower-protein infant formula and follow-on formula (1.77 and 2.2 g intact protein/100 kcal) in the first year of life had a lower weight at age 2 years than infants fed higher-protein formulae (2.9 and 4.4 g intact protein/100 kcal) (16). At 24 months, WAZ scores in the lower-protein formula group were not significantly different than those of the breastfed reference group, which is in line with the results of the current study. Follow-up of the subjects through 6 years of age demonstrated that infants fed the higher-protein formula had a significantly higher BMI and a two-fold higher risk of obesity and excess body fat at age 6 years than infants fed the lower-protein formula (17, 51). This evidence suggests that reducing protein content in infant formula may contribute to decreased childhood obesity risk. Along these lines, a recent position paper on strategies to prevent childhood obesity from the Global Federation of International Societies of Pediatric Gastroenterology, Hepatology, and Nutrition identified infant feeding without excessive protein supply as a key intervention that should be further promoted (52). Although the present study was not designed to assess longer-term obesity risk, weight gain in the formula-fed infants tracked very closely with both the WHO median and the breastfed group, as indicated by the upper and lower limits of the 95% CIs for the estimated difference in WAZ remaining within ± 0.5SD throughout the study period. This suggests that the formula-fed infants in the present study have a weight gain pattern that could be expected to minimize later obesity risk. In addition, the pHF-W FoF used in this study has been associated in a high-risk population with reduced prevalence of certain allergic diseases at certain time points until adulthood (53), which may be an additional important health benefit.

The study demonstrated the safety and suitability of the new pHF-W FoF as evidenced by comparable GI tolerance scores along with similarity in the incidence of severe AEs between the formula-fed and breastfed infants over both the 0−4 months and 4−12 months’ time periods. Of note, the incidence of allergy-related AEs such as atopic dermatitis and cow’s milk allergy were not significantly different between formula-fed and breastfed infants. Further, protein biomarker and amino acid concentrations at age 180 days as measured in the formula-fed group were generally within expected ranges. Although all of the sampled infants had plasma concentrations of aspartic acid below the normal range, the mean value in our study (10.2 μmol/L) is above the median value (7 μmol/L) reported previously for infants aged 0–6 months (54). Overall, these results demonstrate that a reduction in the protein content of infant formula in an age-adapted manner that more closely mimics changes observed in breastmilk provides suitable nutrition.

This study has several strengths including being the first trial to our knowledge to evaluate a feeding regimen consisting of pHF-W infant formula and follow-on formula with age-adapted protein content that are closer to breastmilk level and in the case of the FoF, below the current EU legal minimum protein amount with regard to the specific protein hydrolysate as protein source (35). Previous trials have assessed the growth, safety, and suitability of either pHF-W formulae with higher protein content (36, 43–45) or lower-protein formulae with intact proteins (18, 20). The sample size was powered to account for a smaller growth rate in boys than in girls compared to the WHO standards, although this trend was not observed in the current study. The primary outcome of weight gain velocity was an objective measurement recorded in a standardized fashion by study staff, minimizing the potential for bias in outcome ascertainment. Further, GI tolerance was assessed *via* the IGSQ which has been validated by two separate research groups as a suitable tool for assessing tolerability of infant feeding regimens (40, 55). We deliberately did not include a randomized control group of infants consuming standard (intact protein) formula because our objective was to demonstrate growth comparable to “ideal” infant growth patterns represented by the WHO curves and a breastfed reference group. This study also has limitations. For example, the study FoF was introduced at age 4 months, which is consistent with the legal definition of FoF in Europe (i.e., food used by infants when complementary feeding is introduced), whereas in typical practice FoF is usually introduced at age 6 months. Although this may limit the overall generalizability of our results to populations with more typical feeding patterns, it is nevertheless reassuring that safety and suitability were demonstrated in infants younger than those who might normally consume this formula. Despite the similarity in infant characteristics in the formula-fed and breastfed groups, parental demographics differed between groups in terms of education level and smoking habits, which may have impacted study results. In addition, while offering vouchers for formula or complementary foods to the breastfeeding group was well-intended from both a scientific and ethical standpoint, this might have influenced the eating habits of this group, making it less representative of the general population. A substantial proportion of formula-fed infants had major protocol deviations related to feeding regimen compliance, leading to exclusion from the FAS population. Additionally, this study was conducted in five centers across France and may not be fully generalizable to other regions or cultures with different feeding practices. Finally, while infants were followed through 12 months of age, a longer follow-up period would have allowed an evaluation of the effect of the lower-protein formula on childhood obesity risk.

In conclusion, this study demonstrated that an infant feeding regimen consisting of a standard pHF-W infant formula for the first four months followed by the new pHF-W follow-on formula with age-adapted protein content from age 4−12 months supports healthy infant growth and is safe and well-tolerated when compared with the WHO growth standards and breastfed infants. While breastfeeding remains the optimal source of nutrition for infants, a follow-on formula with a protein content comparable to that of human milk represents a safe and tolerable option for formula-fed infants.

## Data availability statement

The datasets presented in this article are not readily available because further sharing of data was not included in the protocol or informed consent form signed by study participants. Requests to access the datasets should be directed to NH, nicholaspaul.hays@nestle.com.

## Ethics statement

The studies involving human participants were reviewed and approved by the Comité de Protection des Personnes SUD-EST IV and the Agence Nationale de Sécurité du Medicament et des Produits du Santé. Written informed consent to participate in this study was provided by the participants’ parents/legal guardian/next of kin.

## Author contributions

LD was responsible for all statistical analyses. CB and NH wrote the first draft of the manuscript and revised it critically for important intellectual content based on feedback from the other authors. All authors made substantial contributions to conception and design, acquisition of data, interpretation of data, and provided final approval of the version to be published.
