# Supplementary material for: A new partially hydrolyzed whey-based follow-on formula with age-adapted protein content supports healthy growth during the first year of life
Source: Front Pediatr. 2022 Sep 28;10:937882. doi: 10.3389/fped.2022.937882 (PMC9554543; doi:10.3389/fped.2022.937882)
Supplement: Supplementary file 1 [file Data_Sheet_1.docx]

Supplementary Material

# Supplementary Tables

**Supplemental Table 1.** Energy and macronutrient composition of the study formulae.

|  | **Infant Formula** | | **Follow-on Formula** | |
| --- | --- | --- | --- | --- |
| **Parameter** | **Target value** | **Analytical value** | **Target value** | **Analytical value** |
| Energy (kcal/100g) | 507.4 | 510.4 | 486.2 | 491.6 |
| Proteins (g/100kcal) | 1.90 | 1.87 | 1.60 | 1.54 |
| Lipids  (g/100kcal) | 5.12 | 5.09 | 4.73 | 4.72 |
| Carbohydrates  (g/100kcal) | 11.57 | 11.82 | 12.76 | 13.12 |

**Supplemental Table 2.** Serum biomarkers of protein status and plasma amino acid concentrations, at 4 and 6 months (formula-fed infants only)

|  |  | **Female** | | | **Male** | | | **Total** | | | |
| --- | --- | --- | --- | --- | --- | --- | --- | --- | --- | --- | --- |
|  | **Age** | ***n*** | **Mean ± SD** | ***n* (%) below normal** | ***n*** | **Mean ± SD** | ***n* (%) below normal** | ***n*** | **Mean ± SD** | ***n* (%) below normal** |  |
| Albumin (g/L) | 4 months | 23 | 41.41 ± 2.61 | 0 (0%) | 45 | 41.02 ± 2.25 | 0 (0%) | 68 | 41.15 ± 2.37 | 0 (0%) |  |
|  | 6 months | 24 | 41.60 ± 2.47 | 0 (0%) | 38 | 40.93 ± 4.20 | 2 (5.3%) | 62 | 41.19 ±3.62 | 2 (3.2%) |  |
| BUN (mmol/L) | 4 months | 23 | 2.90 ± 0.57 | 2 (8.7%) | 45 | 2.59 ± 0.57 | 9 (20.0%) | 68 | 2.69 ± 0.59 | 11 (16.2%) |  |
|  | 6 months | 23 | 2.33 ± 0.79 | 12 (50.0%) | 39 | 2.44 ± 0.80 | 14 (35.9%) | 62 | 2.40 ± 0.79 | 26 (41.3%) |  |
| Prealbumin (g/L) | 4 months | 23 | 0.16 ± 0.03 | 3 (13.0%) | 45 | 0.16 ± 0.03 | 4 (8.9%) | 68 | 0.16 ± 0.03 | 7 (10.3%) |  |
|  | 6 months | 24 | 0.16 ± 0.02 | 1 (4.2%) | 38 | 0.16 ± 0.04 | 1 (2.6%) | 62 | 0.16 ± 0.03 | 2 (3.2%) |  |
| Alanine (µmol/L) | 6 months | 17 | 525.24 ± 155.71 | 0 (0%) | 13 | 502.32 ± 143.01 | 0 (0%) | 30 | 515.31 ± 148.23 | 0 (0%) |  |
| Arginine (µmol/L) | 6 months | 17 | 136.07 ± 36.16 | 0 (0%) | 13 | 142.80 ± 28.38 | 0 (0%) | 30 | 138.98 ± 32.65 | 0 (0%) |  |
| Asparagine (µmol/L) | 6 months | 17 | 725.52 ± 10.60 | 0 (0%) | 13 | 726.35 ± 6.16 | 0 (0%) | 30 | 725.88 ± 8.83 | 0 (0%) |  |
| Aspartic acid (µmol/L) | 6 months | 17 | 9.70 ± 1.89 | 17 (100%) | 13 | 10.90 ± 2.31 | 13 (100%) | 30 | 10.22 ± 2.13 | 30 (100%) |  |
| Citrulline (µmol/L) | 6 months | 17 | 27.55 ± 11.50 | 1 (5.9%) | 13 | 31.02 ± 12.07 | 0 (0%) | 30 | 29.05 ± 11.68 | 1 (3.3%) |  |
| Cysteine (µmol/L) | 6 months | 17 | 79.15 ± 17.03 | N/A | 13 | 81.14 ± 24.36 | N/A | 30 | 80.01 ± 20.17 | N/A |  |
| Glutamic acid (µmol/L) | 6 months | 17 | 18.61 ± 4.01 | 2 (11.8%) | 13 | 18.82 ± 4.67 | 1 (7.7%) | 30 | 18.70 ± 4.23 | 3 (10.0%) |  |
| Glycine (µmol/L) | 6 months | 17 | 257.88 ± 32.17 | 0 (0%) | 13 | 249.42 ± 43.12 | 0 (0%) | 30 | 254.21 ± 36.86 | 0 (0%) |  |
| Histidine (µmol/L) | 6 months | 17 | 103.08 ± 19.52 | 0 (0%) | 13 | 120.75 ± 20.24 | 0 (0%) | 30 | 110.73 ± 21.43 | 0 (0%) |  |
| Homocysteine (µmol/L) | 6 months | 17 | 280.87 ± 91.02 | N/A | 13 | 319.32 ± 189.64 | N/A | 30 | 297.53 ± 140.81 | N/A |  |
| Isoleucine (µmol/L) | 6 months | 17 | 100.38 ± 33.58 | 0 (0%) | 13 | 105.42 ± 22.58 | 0 (0%) | 30 | 102.56 ± 28.97 | 0 (0%) |  |
| Leucine (µmol/L) | 6 months | 17 | 155.34 ± 43.96 | 0 (0%) | 13 | 165.77 ± 34.15 | 0 (0%) | 30 | 159.86 ± 39.70 | 0 (0%) |  |
| Lysine (µmol/L) | 6 months | 17 | 229.12 ± 52.25 | 0 (0%) | 13 | 265.15 ± 61.42 | 0 (0%) | 30 | 244.73 ± 58.28 | 0 (0%) |  |
| Methionine (µmol/L) | 6 months | 17 | 47.26 ± 16.12 | 0 (0%) | 13 | 52.49 ± 14.97 | 0 (0%) | 30 | 49.53 ± 15.59 | 0 (0%) |  |
| Ornithine (µmol/L) | 6 months | 17 | 129.58 ± 29.54 | 0 (0%) | 13 | 140.92 ± 34.77 | 0 (0%) | 30 | 134.49 ± 31.85 | 0 (0%) |  |
| Phenylalanine (µmol/L) | 6 months | 17 | 95.89 ± 18.88 | 0 (0%) | 13 | 104.10 ± 15.48 | 0 (0%) | 30 | 99.45 ± 17.69 | 0 (0%) |  |
| Proline (µmol/L) | 6 months | 17 | 211.09 ± 70.95 | 0 (0%) | 13 | 231.47 ± 68.47 | 0 (0%) | 30 | 219.92 ± 69.44 | 0 (0%) |  |
| Serine (µmol/L) | 6 months | 17 | 169.69 ± 24.04 | 0 (0%) | 13 | 185.53 ± 37.92 | 0 (0%) | 30 | 176.55 ± 31.27 | 0 (0%) |  |
| Taurine (µmol/L) | 6 months | 17 | 74.21 ± 18.92 | 0 (0%) | 13 | 77.42 ± 22.01 | 0 (0%) | 30 | 75.60 ± 20.01 | 0 (0%) |  |
| Threonine (µmol/L) | 6 months | 17 | 174.44 ± 40.71 | 0 (0%) | 13 | 203.12 ± 62.84 | 0 (0%) | 30 | 186.87 ± 52.51 | 0 (0%) |  |
| Tryptophan (µmol/L) | 6 months | 17 | 224.47 ± 38.80 | 0 (0%) | 13 | 246.38 ± 45.16 | 0 (0%) | 30 | 233.96 ± 42.38 | 0 (0%) |  |
| Tyrosine (µmol/L) | 6 months | 17 | 124.84 ± 39.03 | 0 (0%) | 13 | 125.40 ± 26.46 | 0 (0%) | 30 | 125.08 ± 33.62 | 0 (0%) |  |
| Valine (µmol/L) | 6 months | 17 | 224.97 ± 87.57 | 2 (11.8%) | 13 | 249.52 ± 45.82 | 0 (0%) | 30 | 235.61 ± 72.48 | 2 (6.7%) |  |

BUN = blood urea nitrogen; N/A = not available (reference range not provided by laboratory).

**Supplemental Table 3.** Reported dietary intake from complementary foods and liquids by feeding group and time period (FAS population).

| **Parameter** | **Visit** | **Breastfed** | | **Formula-Fed** | |
| --- | --- | --- | --- | --- | --- |
|  |  | ***n*** | **Mean ± SD** | ***n*** | **Mean ± SD** |
| Energy (kcal/d) | 5 mo | 49 | 113.7 ± 174.5 | 70 | 112.2 ± 143.0 |
|  | 6 mo | 71 | 189.6 ± 147.3 | 81 | 194.9 ± 151.1 |
|  | 9 mo | 73 | 424.7 ± 178.7 | 81 | 494.9 ± 182.7 |
|  | 12 mo | 70 | 579.8 ± 181.2 | 78 | 606.5 ± 212.7 |
| Carbohydrates (g/d) | 5 mo | 49 | 13.93 ± 13.01 | 70 | 17.75 ± 21.51 |
|  | 6 mo | 71 | 28.76 ± 23.78 | 81 | 30.90 ± 25.41 |
|  | 9 mo | 73 | 61.80 ± 28.45 | 81 | 73.34 ± 29.42 |
|  | 12 mo | 70 | 80.85 ± 28.45 | 78 | 87.92 ± 32.64 |
| Carbohydrates (% energy) | 5 mo | 49 | 56.90 ± 23.57 | 70 | 61.24 ± 23.16 |
|  | 6 mo | 71 | 58.51 ± 18.11 | 81 | 62.16 ± 15.82 |
|  | 9 mo | 73 | 58.17 ± 9.61 | 81 | 59.12 ± 10.53 |
|  | 12 mo | 70 | 55.94 ± 9.63 | 78 | 57.91 ± 8.31 |
| Lipids (g/d) | 5 mo | 49 | 4.96 ± 15.06 | 70 | 3.06 ± 6.16 |
|  | 6 mo | 71 | 5.03 ± 4.80 | 81 | 4.53 ± 4.90 |
|  | 9 mo | 73 | 11.30 ± 7.03 | 81 | 12.75 ± 7.59 |
|  | 12 mo | 70 | 17.26 ± 9.09 | 78 | 16.44 ± 8.37 |
| Lipids (% energy) | 5 mo | 49 | 27.67 ± 24.20 | 70 | 23.06 ± 23.68 |
|  | 6 mo | 71 | 24.80 ± 18.16 | 81 | 19.78 ± 12.21 |
|  | 9 mo | 73 | 23.53 ± 9.04 | 81 | 22.41 ± 8.17 |
|  | 12 mo | 70 | 26.00 ± 9.10 | 78 | 24.06 ± 7.25 |
| Proteins (g/d) | 5 mo | 49 | 2.04 ± 2.53 | 70 | 2.27 ± 3.22 |
|  | 6 mo | 71 | 4.96 ± 4.82 | 81 | 5.40 ± 4.79 |
|  | 9 mo | 73 | 14.10 ± 6.36 | 81 | 16.94 ± 7.12 |
|  | 12 mo | 70 | 19.40 ± 6.10 | 78 | 20.84 ± 8.57 |
| Proteins (% energy) | 5 mo | 49 | 8.76 ± 5.89 | 70 | 8.76 ± 4.95 |
|  | 6 mo | 71 | 10.51 ± 6.03 | 81 | 11.68 ± 5.99 |
|  | 9 mo | 73 | 13.39 ± 3.46 | 81 | 14.21 ± 4.26 |
|  | 12 mo | 70 | 13.82 ± 3.55 | 78 | 13.91 ± 3.20 |
